# Possible Involvement of Opa-Interacting Protein 5 in Adipose Proliferation and Obesity

**DOI:** 10.1371/journal.pone.0087661

**Published:** 2014-02-06

**Authors:** Kana Inoue, Norikazu Maeda, Takuya Mori, Ryohei Sekimoto, Yu Tsushima, Keisuke Matsuda, Masaya Yamaoka, Takayoshi Suganami, Hitoshi Nishizawa, Yoshihiro Ogawa, Tohru Funahashi, Iichiro Shimomura

**Affiliations:** 1 Department of Metabolic Medicine, Graduate School of Medicine, Osaka University, Suita, Osaka, Japan; 2 Department of Organ Network and Metabolism, Graduate School of Medical and Dental Sciences, Tokyo Medical and Dental University, Tokyo, Japan; 3 Department of Molecular Endocrinology and Metabolism, Graduate School of Medical and Dental Sciences, Tokyo Medical and Dental University, Tokyo, Japan; 4 Department of Metabolism and Atherosclerosis, Graduate School of Medicine, Osaka University, Suita, Osaka, Japan; University of Minnesota - Twin Cities, United States of America

## Abstract

Obesity is an epidemic matter increasing risk for cardiovascular diseases and metabolic disorders such as type 2 diabetes. We recently examined the association between visceral fat adiposity and gene expression profile of peripheral blood cells in human subjects. In a series of studies, Opa (Neisseria gonorrhoeae opacity-associated)-interacting protein 5 (OIP5) was nominated as a molecule of unknown function in adipocytes and thus the present study was performed to investigate the role of OIP5 in obesity. Adenovirus overexpressing Oip5 (Ad-Oip5) was generated and infected to 3T3-L1 cells stably expressing Coxsackie-Adenovirus Receptor (CAR-3T3-L1) and to mouse subcutaneous fat. For a knockdown experiment, siRNA against Oip5 (Oip5-siRNA) was introduced into 3T3-L1 cells. Proliferation of adipose cells was measured by BrdU uptake, EdU-staining, and cell count. Significant increase of Oip5 mRNA level was observed in obese white adipose tissues and such increase was detected in both mature adipocytes fraction and stromal vascular cell fraction. Ad-Oip5-infected CAR-3T3-L1 preadipocytes and adipocytes proliferated rapidly, while a significant reduction of proliferation was observed in Oip5-siRNA-introduced 3T3-L1 preadipocytes. Fat weight and number of adipocytes were significantly increased in Ad-Oip5-administered fat tissues. Oip5 promotes proliferation of pre- and mature-adipocytes and contributes adipose hyperplasia. Increase of Oip5 may associate with development of obesity.

## Introduction

Obesity, especially visceral fat-accumulated obesity, is an important aspect of the metabolic syndrome, diabetes, and atherosclerosis [1]. In the Human Body Map project [2], our group for the first time provided evidence for the endocrine function of adipose tissue [3]. We also discovered adiponectin among human adipose tissue cDNAs in this project [4]. Clinical and experimental evidences indicate that adipocytes produce various cytokines and chemokines, which we named adipocytokines, and that the obesity-induced dysregulation of adipocytokines contributes to the development of the metabolic syndrome.

Our group reported recently the association between visceral fat adiposity and gene expression profile of peripheral blood cells in human subjects [5], [6]. We hypothesized that the visceral fat status may affect and reflect the gene expression profile in peripheral blood cells. In a series of exploratory research studies, we searched for genes of unknown function in adipocytes and adipose tissues by comparing the cDNA microarray-based gene expression patterns of mouse adipose tissues and human peripheral blood cells. Our investigation identified several genes among them and present study focused on Opa (Neisseria gonorrhoeae opacity-associated)-interacting protein 5 (OIP5). Opa proteins are a family of outer membrane proteins involved in gonococcal adherence to and invasion of human cells. OIP5 was identified as one of proteins interacting with Opa by the yeast two-hybrid system [7]. To date, there are only around 10 reports for OIP5 and most of papers show the association of OIP5 and cancer. These papers demonstrated that OIP5 is highly expressed in cancer cells and plays a crucial role of mitosis [8]–[13].

To our knowledge, there is virtually no information on the role of OIP5 in obesity and adipose tissues. We herein investigated the significance of OIP5 in adipose tissues, especially focusing on adipose proliferation.

## Materials and Methods

### Animals

Male C57BL/6N mice and *ob/ob* mice were obtained from the Charles River Japan Inc. (Kanagawa, Japan) and maintained at 22°C under a 12∶12-h light–dark cycle (lights on from 7∶00 to 19∶00). For analysis of tissue distribution, male C57BL/6N mice were euthanized by bleeding from the inferior vena cava under anesthesia after 12 hrs of fasting, and various tissue samples were excised at 12 weeks of age. For obese model mice study, C57BL/6N and *ob/ob* mice were analyzed at 16 weeks of age under regular chow (MF; Oriental Yeast, Osaka, Japan). For the diet-induced obesity (DIO) study, 8-week-old male C57BL/6N mice were fed with either regular chow diet (MF; Oriental Yeast, Osaka, Japan) or high-fat and high-sucrose (HF/HS) diet (F2HFHSD; Oriental Yeast, Osaka, Japan) for 16 weeks. Mice were euthanized under feeding condition, epididymal white adipose tissues (WAT) were excised at 24 weeks of age. In all experiments, mice were anesthetized with an intraperitoneal injection of a mixture of medetomidine (0.3 mg/kg body weight), midazolam (4 mg/kg body weight) and butorphanol tartrate (5 mg/kg body weight). The experimental protocols were approved by the Ethics Review Committee for Animal Experimentation of Osaka University School of Medicine. This study also conforms to the Guide for the Care and Use of Laboratory Animals published by the US National Institutes of Health.

### Fractionation of WAT

WAT were minced in Krebs-Ringer buffer containing 120 mmol/L NaCl, 4 mmol/L KH_2_PO_4_, 1 mmol/L MgSO_4_, 1 mmol/L CaCl_2_, 10 mmol/L NaHCO_3_, 30 mmol/L HEPES, 20 mmol/L adenosine, and 4% (wt/vol) bovine serum albumin (Calbiochem, San Diego, CA). Tissue suspensions were centrifuged at 500×*g* for 5 minutes to remove erythrocytes and leukocytes. Collagenase was added to a final concentration of 2 mg/mL and suspensions were incubated at 37°C for 20 minutes under shaking. The cell suspension was filtered through a 250 µm filter and then spun at 300×*g* for 1 minute to separate floating mature adipocytes fraction (MAF) from the stromal vascular cell fraction (SVF) pellet. Such fractioning and washing procedure were repeated twice with Krebs-Ringer buffer. Finally, both fractions were washed with phosphate buffered saline (PBS) and subjected to quantitative real-time polymerase chain reaction (PCR).

### Cell Cultures

Maintenance of 3T3-L1 and differentiation of 3T3-L1 cells were described previously [14]. Briefly, 3T3-L1 cells were maintained in Dulbecco’s modified Eagle medium (DMEM) containing 10% fatal calf serum (FCS) and differentiated with the induction media (10% FCS+DMEM supplemented with 5 µg/mL of insulin, 0.5 mmol/L of 1-methyl-3-isobutyl-xanthin, and 1 µmol/L of dexamethasone) on 48 hours after reaching confluence. On day 2 after induction, media was changed to maintenance media (10% FCS+DMEM). For 3T3-L1 cells stably expressing Coxsackie-Adenovirus Receptor (CAR-3T3-L1), 5 µg/mL of insulin was added to maintenance media (10% FCS+DMEM). On day 9 after the induction of differentiation, 3T3-L1 adipocytes were treated with or without 10 ng/mL of tumor necrosis factor-α (TNF-α), 5 µg/mL of insulin, and 50 µM of H_2_O_2_ for 24 hours, and thus cells were harvested and subjected to quantitative real-time PCR. On day 5 after differentiation, 3T3-L1 adipocytes were treated with 1 µM of pioglitazone (Pio) and rivoglitazone (Rivo), synthetic peroxisome proliferator-activated receptor-γ (PPARγ) ligands, for 24 hours, and thus cells were harvested and subjected to quantitative real-time PCR.

### Introduction of siRNA

When 3T3-L1 preadipocytes were 70–80% confluent, cells were transfected with siRNA for Oip5 (Oip5-siRNA) (forward sequence 5′-GGG UAG CCU UAA ACU UAC ATT-3′ and reverse sequence 5′-UGU AAG UUU AAG GCU ACC CTG-3′, QIAGEN, Valencia, CA) by using lipofectamine-2000 (Life technology, Carlsbad, CA, USA), according to the protocol recommended by the manufacturer. Allstars negative control siRNA (QIAGEN) was used as a control (Cont-siRNA). The transfected cells were incubated for 24 hrs and then reseeded to match the number of 3T3-L1 preadipocytes between Oip5-siRNA and Cont-siRNA groups. 3T3-L1 preadipocytes were harvested at 24 or 72 hrs after reseeding and subjected to quantitative real-time PCR (Figure S1). Bromodeoxyuridine (BrdU) was added at 2 hrs prior to harvest and was measured by Microplate reader SH-9000 (Corona Electric, Ibaraki, Japan). Cell number was counted at 24, 48 or 72 hrs after reseeding.

### Construction and Preparation of Adenovirus Expressing Oip5

Full-length cDNA of Oip5 from mouse colon were subjected to RT-PCR by using Pfu DNA polymerase (Promega, Madison, WI) with primers containing a restriction enzyme (XhoI and KpnI) cutting site at the end. Primer sets were as follows: 5′-AGGGTACCACCATGGCGACTCTCTCGCGCCGCAG-3′ and 5′-CCGCTCGAGTTACAGGATCTTTGGTGATGCTGTTAACC-3′. The amplicons were cloned into pENTRTM1A vector (Life technology, Carlsbad, CA, USA) using restriction enzyme sites. After confirmation of correct sequences, the gene encoding Oip5 in pENTRTM1A vector was transferred into the adenoviral expression vector (pAd/CMV/V5-DEST; Life technology, Carlsbad, CA, USA) by recombination following the manufacturer’s instructions. The resultant pAd/CMV plasmid containing the target gene was linearized by PacI digestion and were transfected into 293A cells by lipofectamine-2000 (Life technology, Carlsbad, CA, USA). On day 2 after transfection, the 293A cells were passaged and were cultivated until 80% of cells became detached. The cell suspension was then frozen and thawed three times. After centrifugation at 1,750×*g* for 15 min, the supernatant was used as the gene expression adenoviral preparation (Ad-Oip5). The titers for the adenoviral preparation were around 5×10^8^ plaque forming units (pfu)/mL. The adenovirus expressing β-galactosidase (Ad-βgal) was used as a control.

### Infection of Adenovirus into Preadipocytes

To obtain the efficient transduction by adenovirus, CAR-3T3-L1 cells were used in the adenoviral study [15], [16]. When cells were 70–80% confluent, CAR-3T3-L1 preadipocytes were infected with Ad-Oip5 or Ad-βgal at 5.0 multiplicity of infection (MOI) (Figure S2). The medium was changed to remove the uninfected adenovirus at 24 hrs from adenovirus infection, and cells were reseeded to match the number of cells between Ad-Oip5 and Ad-βgal groups. CAR-3T3-L1 preadipocytes were collected at 24 or 72 hrs after reseeding, subjected to quantitative real-time PCR. BrdU was added at 2 hrs prior to harvest and was measured by Microplate reader SH-9000 (Corona Electric, Ibaraki, Japan). Cell number of CAR-3T3-L1 preadipocytes was measured by a cell counter.

### Infection of Adenovirus into Adipocytes

On day 5 after differentiation into adipocytes, CAR-3T3-L1 adipocytes were infected with Ad-Oip5 or Ad-βgal at 5.0 MOI (Figure S3). On 24 hrs after adenovirus infection, medium was changed to remove the uninfected adenovirus and CAR-3T3-L1 adipocytes were reseeded to match the number of adipocytes between Ad-Oip5 and Ad-βgal groups. CAR-3T3-L1 adipocytes were harvested at 24 or 72 hrs after reseeding and subjected to quantitative real-time PCR and cell count as above described. CAR-3T3-L1 adipocytes were stained with Oil red O at 24 or 72 hrs after reseeding and measured by Microplate reader SH-9000 (Corona Electric, Ibaraki, Japan).

For immunofluorescence microscopy analysis, the adenovirus-transfected CAR-3T3-L1 adipocytes were reseeded on 2 chamber dishes at 24 hrs after adenovirus infection and subjected to immunostaining at 24 hrs after reseeding. Proliferating CAR-3T3-L1 adipocytes were detected by using Alexa 488 conjugated Click-iT EdU Imaging Kits (Life technology, Carlsbad, CA, USA) according to the protocol recommended by the manufacturer. CAR-3T3-L1 adipocytes were also stained with a rabbit anti-fatty acid binding proteins (FABP4) antibody (Catalog No. #3544, Cell signaling, Danvers, USA) as the 1^st^-antibody and a goat anti-rabbit IgG conjugated Alexa 594 (Life Technologies, Gaithersburg, MD) as the 2^nd^-antibody. Nuclei were stained with 4′,6-diamidino-2-phenylindole (DAPI). We analyzed images by HS All-in-one Fluorescence Microscope BZ-9000 (Keyence, Osaka, Japan) and Confocal laser scanning microscopy FLUOVIEW FV1000-D (Olympus Corporation, Tokyo, Japan).

### Quantitative Real-time PCR Analysis

Isolation of total RNA and production of cDNA were performed as described previously [17]. Real-time PCR was performed on the ViiATM 7 real-time PCR system (Life technologies, Carlsbad, CA, USA) using the THUNDERBIRD TM qPCR Mix (TOYOBO, Osaka, Japan) according to the manufacturer’s instructions. For quantitative precision, the same amount of total RNA was consistently used for each expression analysis and the expression level of each gene was normalized by the mRNA level of a housekeeping gene, ribosomal protein, large, P0 (Rplp0/36B4). Primers used in this study were as follows: mouse Oip5, 5′-AGA GGC CAT TTC TGC CTT TC-3′ and 5′-CAG CTC TGC AAT CTT TTC TGG-3′; mouse Rplp0/36B4, 5′- AAG CGC GTC CTG GCA TTG TCT-3′ and 5′- CCG CAG GGG CAG CAG TGG T-3′.

### The in vivo Adenovirus-mediated Gene Transfer of Oip5 to Mouse Adipose Tissues

Eight-week-old male C57BL/6N mice were made incisions at approximately 1 cm in bilateral groins and the 25 µL (2.5×10^9^ pfu/mL) of Ad-βgal and Ad-Oip5 were injected in each side of the subcutaneous fat at the groin, respectively. Animals were killed and analyzed on day 4, 11, and 21 after adenovirus administration. Subcutaneous fat tissues of the upper side from femoral artery were weighed by microbalance and stained with hematoxylin and eosin (H&E). The number of nuclei was calculated by using BZ analyzer in Hybrid Cell Count mode (Keyence, Osaka, Japan). For diet-induced obesity (DIO) study, male C57BL/6N mice were fed with HF/HS diet from 6 weeks of age and locally administered with Ad-Oip5 or Ad-βgal to both subcutaneous WAT at 8 weeks of age, according to the same procedure as described above. After adenovirus administration, mice were analyzed at 13 weeks of age.

### Time-lapse Imaging

For living adipocytes imaging, time-lapse microscopic technique was introduced by using the confocal laser scanning microscopy FLUOVIEW FV1000-D (Olympus Corporation, Tokyo, Japan). 3T3-L1 adipocytes were reseeded on 2 chamber dishes at 24 hrs after adenovirus infection and on day 7 after differentiation subjected to time-lapse imaging from 24 to 44 hrs after reseeding. A sealed dish of CAR-3T3-L1 adipocytes was placed in an acrylic resin box in which temperature was maintained at 37°C on the sample stage, and time-lapse images were captured every 6 min. The series of captured images were converted into a movie using MetaMorph® software (Molecular Devices, Sunnyvale, California).

### Statistical Analysis

All values are presented as means ±1 SE. Statistical analysis was performed by using one-factor ANOVA and the unpaired Student’s t-test. P values less than 0.05 were considered statistically significant.

## Results

### Changes of Oip5 mRNA Expression in Obese Adipose Tissues

Signal intensity levels of OIP5 mRNA in peripheral blood cells positively correlated with the estimated visceral fat area (eVFA) in human subjects (range of BMI, 25.4–51.2 kg/m^2^; range of eVFA, 80–386 cm^2^) (Figure S4). The role of Oip5 in adipocytes and adipose tissues was analyzed by using mice and cells. Firstly, tissue distribution for Oip5 mRNA expression level was examined in lean control C57BL/6N (B6) mice (Figure S5). Oip5 mRNA was abundantly detected in testis and was highly expressed in spleen, colon, and small intestine. In contrast to these tissues, Oip5 mRNA level was low in skeletal muscle, aorta, and liver. Oip5 mRNA was also detected in epididymal (Epi) and subcutaneous (Sub) white adipose tissue (WAT).

**Figure 1:**
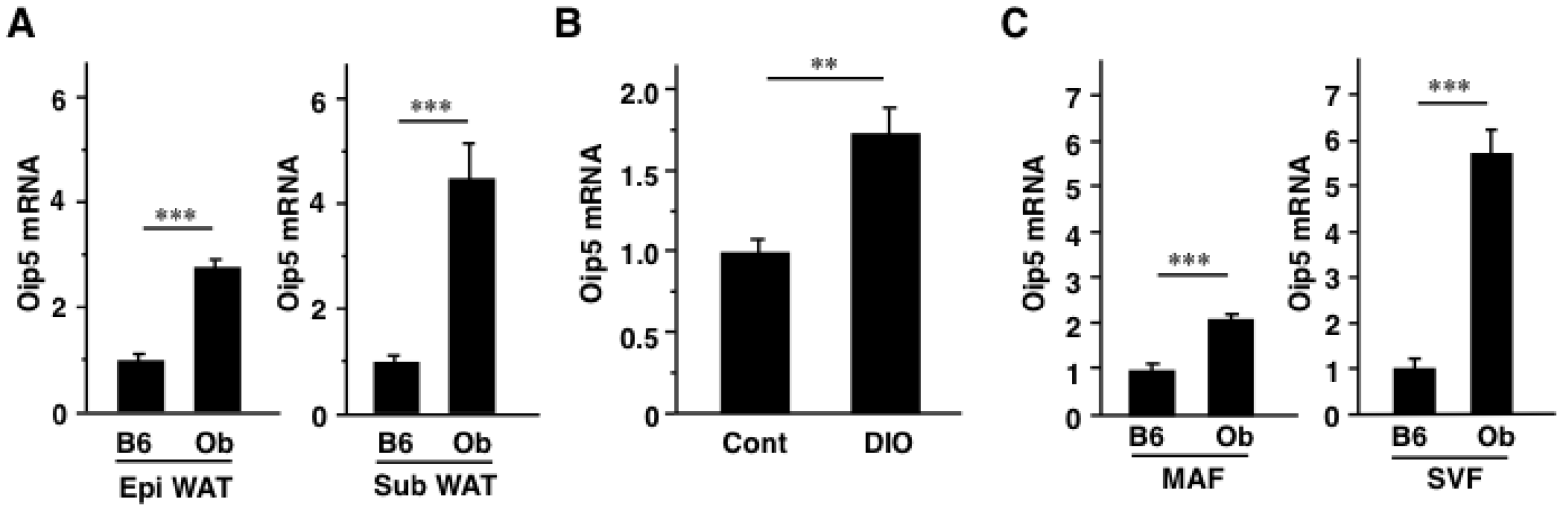
The mRNA expressions of Oip5 in obese adipose tissues. A, Adipose Oip5 mRNA levels in obese model mice. Oip5 mRNA levels of epididymal (Epi) and subcutaneous (Sub) white adipose tissues (WAT) were examined in C57BL/6N (B6) and *ob/ob* (Ob) mice at 16 weeks of age. B, Change of adipose Oip5 mRNA level in diet-induced obesity (DIO). C57BL/6J mice were fed with regular chaw diet or high-fat/high-sucrose (HF/HS) diet from 8 weeks of age and were analyzed at 16 weeks of age. C, Oip5 mRNA level in the fractionated WAT. WAT of B6 and Ob mice at 16 weeks of age were separated into mature adipocytes fraction (MAF) and stromal vascular fraction (SVF) as described in Materials and Methods section. Oip5, Opa-interacting protein 5; Cont, C57BL/6N mice fed with regular chow. Values are mean ± SE; n = 6 for each group. **P<0.01;***P<0.001.

Change of Oip5 mRNA level was next examined in obese mice. Adipose Oip5 mRNA level was significantly higher in *ob/ob* (Ob) mice than in B6 mice (Figure 1A). Oip5 mRNA level of WAT was significantly increased in DIO mice, compared to control mice fed with regular chow (Figure 1B). Next, Oip5 mRNA level was examined in MAF and SVF following fractionation of adipose tissues. Oip5 mRNA levels of MAF and SVF in Ob mice were significantly increased 2.1-fold and 5.7-fold, respectively, compared to B6 mice (Figure 1C).

### Effect of the Overexpression and Knockdown of Oip5 in 3T3-L1 Preadipocytes

**Figure 2:**
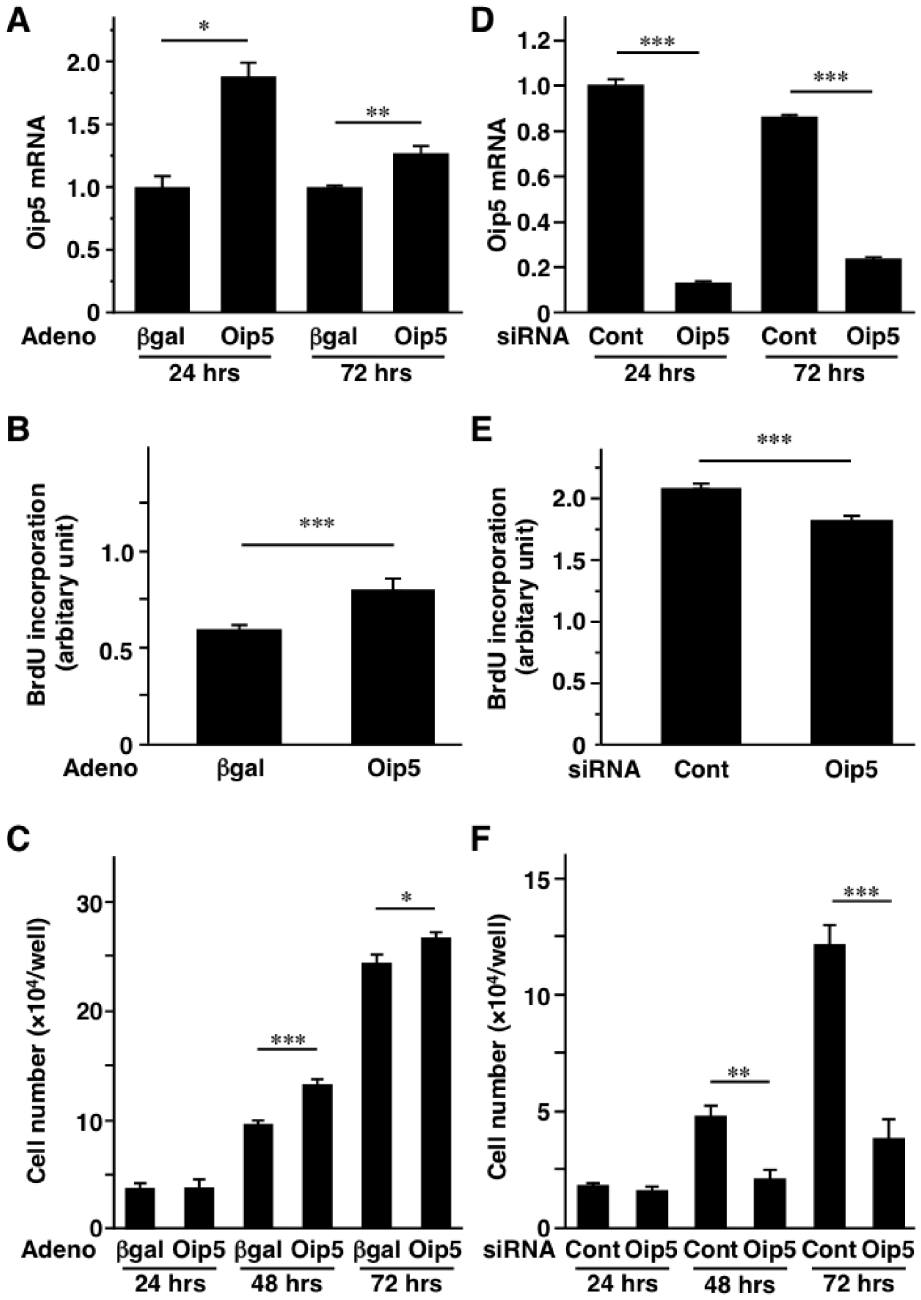
Effect of Oip5 on preadipocytes proliferation. For the overexpression study, 3T3-L1 cells stably expressing Coxsackie-Adenovirus Receptor (CAR)-3T3-L1 were used as described in Materials and Methods section (A to C). For the knockdown experiments, introduction of siRNA was performed in 3T3-L1 preadipocytes as described in Materials and Methods section (D to F). A, Increase of Oip5 mRNA level by adenovirus-mediated Oip5 gene transfer. B, Incorporation of BrdU in the adenovirus-infected CAR-3T3-L1 preadipocytes at 24 hrs after reseeding. C, Number of adenovirus-infected CAR-3T3-L1 preadipocytes at 24, 48, and 72 hrs after reseeding. D, Decrease of Oip5 mRNA level by siRNA for Oip5 at 24 and 72 hrs after reseeding. E, Uptake of BrdU in siRNA-introduced 3T3-L1 preadipocytes at 24 hrs after reseeding. F, Number of siRNA-introduced 3T3-L1 preadipocytes at 24, 48, and 72 hrs after reseeding. Adeno, adenovirus; Oip5, Opa-interacting protein 5; βgal, β-galactosidase; Cont, control. Values are mean ± SE; n = 6 for each group. *P<0.05; **P<0.01;***P<0.001.

As shown in Figure S5, Oip5 expression was highly observed in the tissues where cell proliferation is promoted. Previous reports demonstrated the abundance of Oip5 in cancer cells. These results suggest that Oip5 may be associated with cell proliferation and thus present study focused on the role of Oip5 in adipose proliferation. Adenoviral overexpression and siRNA-mediated suppression of Oip5 were firstly performed in 3T3-L1 preadipocytes (Figure 2), because a remarkable increase of Oip5 mRNA level was observed in obese adipose SVF containing preadipocytes (Figure 1C). Significant increase of Oip5 mRNA level was confirmed in the Ad-Oip5-administered CAR-3T3-L1 preadipocytes on 24 and 72 hrs after reseeding of cells (Figure 2A). Oip5 mRNA level was hardly influenced by the infection of Ad-βgal compared to the non-infected cells (data not shown). Incorporation of BrdU was significantly increased in Ad-Oip5-infected preadipocytes compared to Ad-βgal group (Figure 2B). Number of Ad-Oip5-infected preadipocytes was significantly more than that of Ad-βgal-infected preadipocytes on 48 and 72 hrs after reseeding (Figure 2C).

Next, the effect of Oip5-knockdown on preadipocytes proliferation was examined. Effective knockdown of Oip5 was observed in Oip5-siRNA-introduced 3T3-L1 preadipocytes (Figure 2D). Oip5 mRNA level was scarcely changed by the introductuion of Cont-siRNA compared to non-transfected cells (data not shown). Uptake of BrdU into preadipocytes was significantly reduced in Oip5-siRNA group compared to Cont-siRNA group (Figure 2E). Number of Cont-siRNA-introduced preadipocytes increased properly, while cell number was significantly decreased in Oip5-siRNA-introduced preadipocytes on 48 and 72 hrs after reseeding of cells (Figure 2F). These results indicated that Oip5 should promote preadipocytes proliferation.

### Effect of the Oip5-overexpression in 3T3-L1 Adipocytes

**Figure 3:**
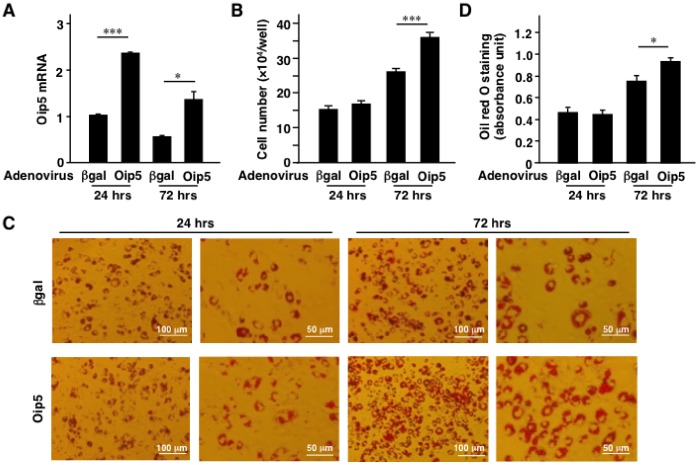
Effect of Oip5 overexpression on adipocytes proliferation. Adenovirus expressing β-galactosidase (βgal) or Oip5 was infected in Coxsackie-Adenovirus Receptor (CAR)-3T3-L1 adipocytes as described in Materials and Methods section. A, Oip5 mRNA level of the adenovirus-infected CAR-3T3-L1 adipocytes at 24 and 72 hrs after reseeding. B, Number of the adenovirus-infected CAR-3T3-L1 adipocytes at 24 hrs and 72 hrs after reseeding. C, Quantification of oil red O staining of the adenovirus-infected CAR-3T3-L1 adipocytes at 24 and 72 hrs after reseeding. D, Oil red O staining images of the adenovirus-infected CAR-3T3-L1 adipocytes at 24 and 72 hrs after reseeding. Oip5, Opa-interacting protein 5; βgal, β-galactosidase. Values are mean ± SE; n = 6 for each group. *P<0.05;***P<0.001.

**Figure 4:**
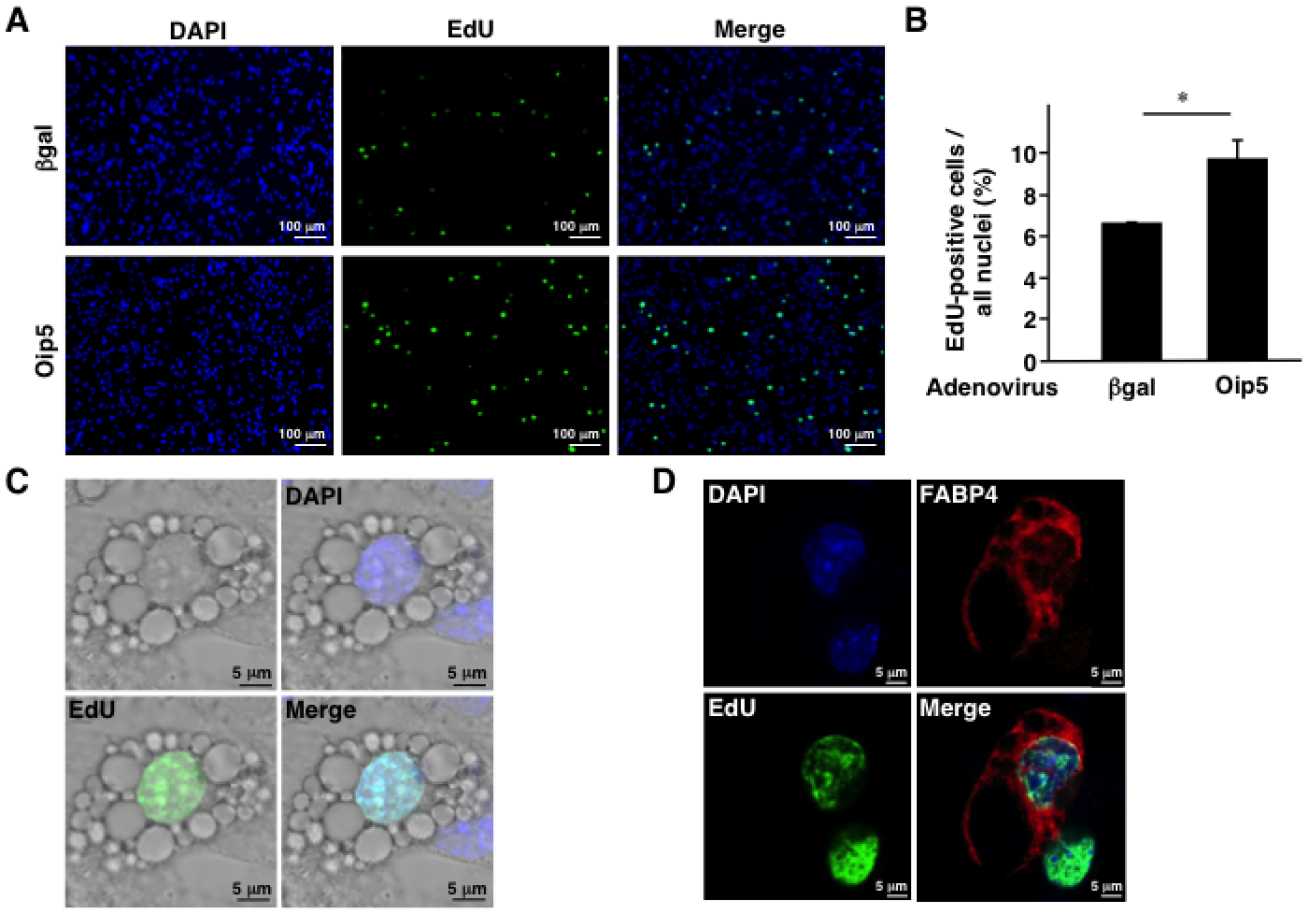
Immunostainings of CAR-3T3-L1 adipocytes overexpressing Oip5. A, Images of EdU staining in the adenovirus-transfected Coxsackie-Adenovirus Receptor (CAR)-3T3-L1 adipocytes. CAR-3T3-L1 adipocytes were stained with 5-ethynyl-2′-deoxyuridine (EdU) and 4′,6-diamidino-2-phenylindole (DAPI) and analyzed by HS All-in-one Fluorescence Microscope BZ-9000. B, Number of EdU-positive CAR-3T3-L1 cells. Shown is the proportion of EdU-positive CAR-3T3-L1 cells to all nuclei. C, Images for EdU staining of CAR-3T3-L1 adipocytes analyzed by the differential interference contrast microscopy. D, CAR-3T3-L1 adipocytes co-staining with EdU and fatty acid binding protein 4 (FABP4). Images were obtained by confocal laser scanning microscopy. Oip5, Opa-interacting protein 5; βgal, β-galactosidase. Values are mean ± SE; n = 3. *P<0.05.

As in Figure 1C, Oip5 mRNA level was also increased in obese MAF, and thus adenoviral overexpression of Oip5 was next conducted in CAR-3T3-L1 adipocytes (Figure 3 and 4). Significant increase of Oip5 mRNA was confirmed in Ad-Oip5-infected CAR-3T3-L1 adipocytes on 24 and 72 hrs after reseeding (Figure 3A). Number of cells was significantly increased by Ad-Oip5 at 72 hrs after reseeding, compared to Ad-βgal (Figure 3B).

Next, Oil red O staining and immunostaining were performed to confirm proliferation of adipocytes. At 72 hrs after reseeding, number of Oil red O-positive cells was significantly increased in Ad-Oip5 group compared to Ad-βgal group (Figure 3C and 3D). The 5-ethynyl-2′-deoxyuridine (EdU)-positive cells were significantly increased in Ad-Oip5 group compared to Ad-βgal group (Figure 4A and 4B). To confirm adipocytes proliferation, the differential interference contrast microscopic technique was performed. EdU-positive nuclei were observed in the differentiated adipocytes (Figure 4C) and 50% of EdU-positive cells were the differentiated adipocytes by the low magnification observations (data not shown). In addition, EdU-positive nuclei were also co-stained with FABP4, a marker of adipocytes (Figure 4D). Moreover, time-lapse imaging showed the cell division of adipocytes (Movie S1 and S2). These results suggested that the differentiated adipocytes could split by adenoviral expression of Oip5.

### Adenovirus-mediated Gene Transfer of Oip5 to in vivo Adipose Tissue

**Figure 5:**
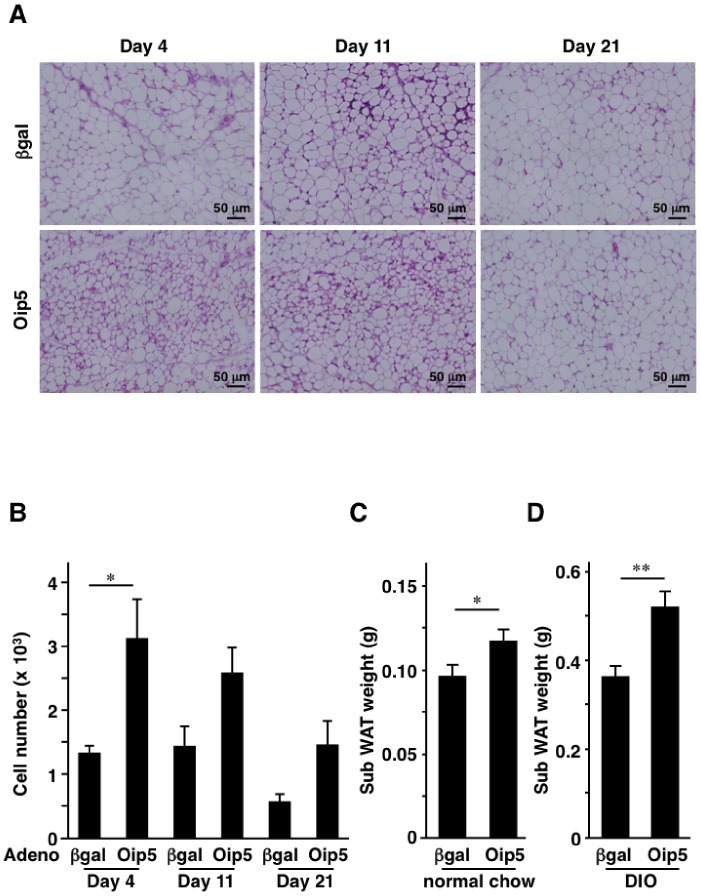
Local adenovirus-mediated gene transfer of Oip5 to in vivo adipose tissues. C57BL/6N mice were injected with 25 µL (2.5×10^9^ pfu/mL) of adenovirus expressing β-galactosidase (βgal) or Oip5 into subcutaneous fat. Mice were analyzed at day 4, 11, and 21 after adenovirus administration. A, Representative hematoxylin and eosin (H&E)-staining section of fat tissues at 4, 11, and 21 days after adenovirus injection. B, Number of cells after adenovirus administration. The number of nucleus was calculated by using BZ analyzer in Hybrid Cell Count mode. C, Subcutaneous fat weights at day 11 after adenovirus administration. Mice were fed with normal chow and were administered with adenovirus at 8 weeks of age. D, Adenovirus-administered subcutaneous fat weights of diet-induced obesity (DIO). C57BL/6N mice were fed with high-fat/high-sucrose (HF/HS) diet from 6 weeks of age and were administered with adenovirus at 8 weeks of age. Mice were analyzed at 13 weeks of age. Oip5, Opa-interacting protein 5; βgal, β-galactosidase. Adeno, adenovirus. Sub WAT, subcutaneous white adipose tissues. Values are mean ± SE; n = 3 (in B), and n = 9–10 (in C and D) for each group. *P<0.05;**P<0.01.

To elucidate the in vivo role of adipose Oip5, B6 mice were locally administered with Ad-Oip5 or Ad-βgal to subcutaneous fat, as described in Materials and Methods section. Figure 5A shows H&E staining section of adenovirus-injected subcutaneous fat tissues. The number of small adipocytes was increased especially on day 4 and day 11 after adenovirus administration (Figure 5A). Higher magnification revealed that multilocular adipocytes, containing 10–20 small lipid droplets, were increased in Ad-Oip5-injected subcutaneous fat tissues on day 11 after adenovirus administration (Figure S6). The number of cells was significantly increased in Ad-Oip5-injected subcutaneous fat tissues compared to Ad-βgal-injection group on day 4 after adenovirus administration (Figure 5B). Such tendency was maintained at day 11 and 21 after adenovirus administration (Figure 5B). Fat weights were significantly heavier in Ad-Oip5 group than in Ad-βgal group on day 11 after adenovirus administration (Figure 5C). Ad-Oip5 injection also increased the weight of subcutaneous WAT in DIO model mice (Figure 5D). In the DIO study, there were no significant differences in the metabolic parameters such as body weight, food intake, and fasting plasma levels of glucose and insulin, between Ad-Oip5- and Ad-βgal-administered mice (data not shown).

### Changes of Oip5 mRNA Level in 3T3-L1 Adipocytes

**Figure 6:**
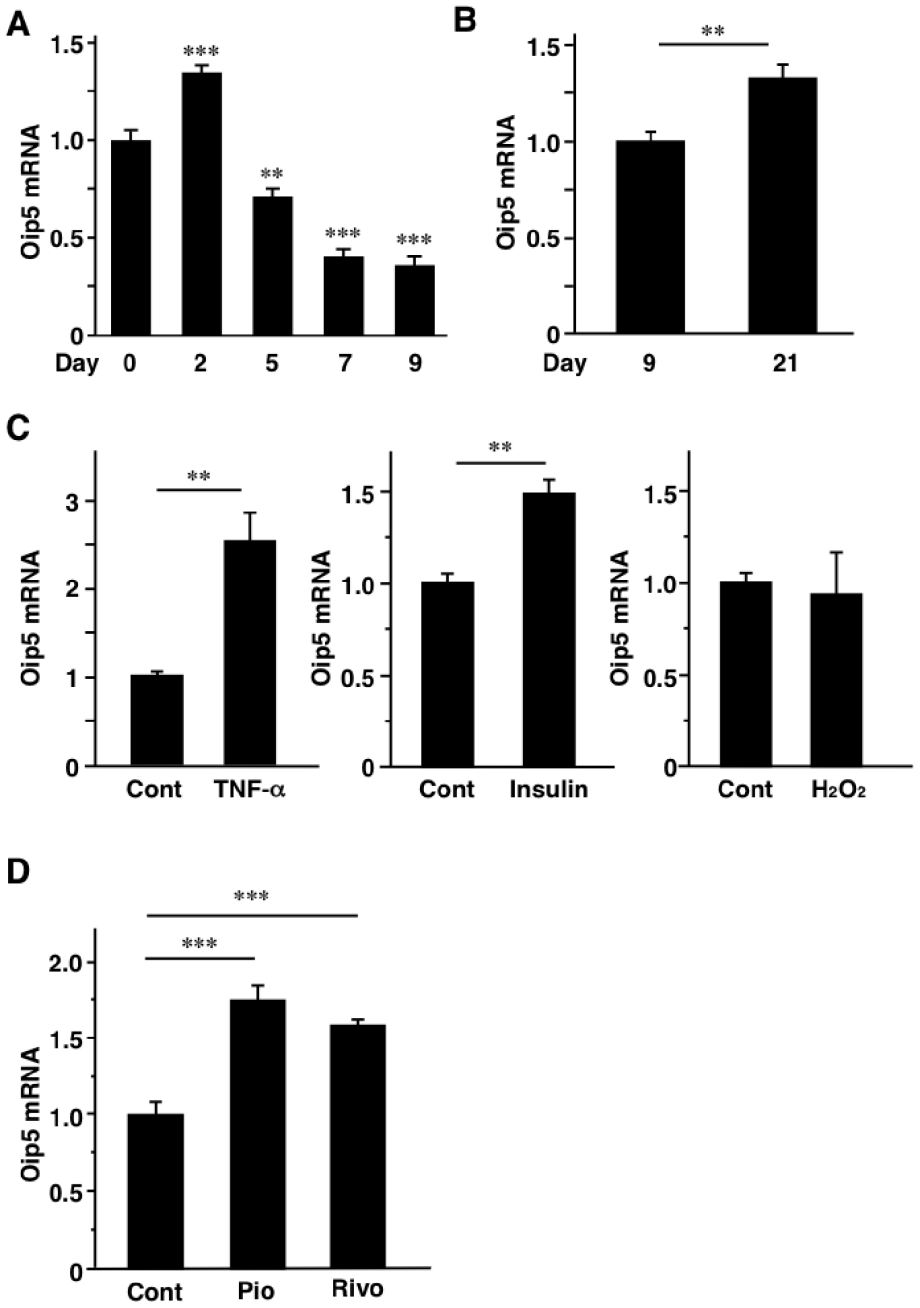
Regulation of Oip5 in 3T3-L1 adipocytes. A, Change of Oip5 mRNA level during 3T3-L1 adipocytes differentiation. **P<0.01;***P<0.001, compared to day 0. B, Oip5 mRNA level of 3T3-L1 adipocytes on day 9 and 21 after the induction of differentiation. C, Effect of tumor necrosis factor-α (TNF-α), insulin and H_2_O_2_ on Oip5 mRNA levels in 3T3-L1 adipocytes. 3T3-L1 adipocytes were treated with or without 10 ng/mL of TNF-α, 5 µg/mL of insulin, and 50 µM of H_2_O_2_ for 24 hrs. D, Effect of peroxisome proliferator-activated receptor-γ (PPARγ) agonists on Oip5 mRNA levels. 3T3-L1 adipocytes were treated with 1 µM of pioglitazone (Pio) and rivoglitazone (Rivo) for 24 hrs. Oip5, Opa-interacting protein 5; TNF-α, tumor necrosis factor-α; Cont, control; Pio, pioglitazone; Rivo, rivoglitazone. Values are mean ± SE; n = 3–6 for each group. **P<0.01;***P<0.001.

Regulation of adipose Oip5 has not been reported and thus it was investigated in 3T3-L1 adipocytes. Oip5 mRNA level peaked on day 2 after induction of 3T3-L1 cells differentiation and gradually decreased during adipocytes differentiation (Figure 6A). However, Oip5 mRNA level was significantly increased on day 21 after differentiation, compared to day 9 (Figure 6B). Next, 3T3-L1 adipocytes were treated with several factors mimicking obese adipose tissues. Treatment with TNF-α and insulin increased Oip5 mRNA levels while Oip5 mRNA level was not changed by H_2_O_2_ administration (Figure 6C). Interestingly, PPARγ ligands, Pio and Rivo, significantly increased Oip5 mRNA level (Figure 6D).

## Discussion

The major findings of the present study were: *(1)* Oip5 mRNA level was significantly increased in obese WAT and such increase was observed in both MAF and SVF. *(2)* Oip5 enhanced the proliferation of both preadipocytes and adipocytes. *(3)* Oip5 increased the number of small adipocytes in subcutaneous fat tissues. *(4)* TNF-α and insulin increased Oip5 mRNA level and PPARγ ligands augmented its mRNA level.

It has been demonstrated that OIP5 may contribute to the growth of cancer. Abundant expression of OIP5 is observed in cancer cells such as esophageal cancer, gastric cancer, colorectal cancer, lung cancer, renal cell carcinoma, and acute myeloid leukemia [8]–[12]. OIP5 was identified among the up-regulated genes in gastric cancer cells by using cDNA microarray analysis [8]. The other group also showed the high expression level of OIP5 in lung and esophageal cancers by using cDNA microarray analysis and furthermore demonstrated that OIP5 expression level was significantly associated with poor prognosis of these patients [10]. Fujita et al clearly showed that OIP5 is one of essential molecules for centromere function, indicating that OIP5 contributes to mitosis [13]. Present study for the first time showed the significance of Oip5 in the proliferation of both preadipocytes and adipocytes. However, it will be needed to clarify the potential contribution of adipose Oip5 to cell cycle by generating retrovirus-mediated gene transfer system.

In the development of obesity, excess of energy results in the increase of energy storage in cells, mainly in adipocytes. Increased fat storage in differentiated adipocytes results in enlarged adipocytes. However, there are limitations of fat cell volume, i.e. size of adipocytes reaches plateau while body fat mass severely increased. Arner’s group demonstrated that new adipocytes constantly replaced the lost adipocytes, such that approximately 10% of adipocytes are annually replaced in human subcutaneous adipose tissues [18]. Adipocyte progenitor cells (preadipocytes) locate in the perivascular compartment of WAT and are capable of differentiation into mature adipocytes [19]–[21]. Accumulating evidences postulate ‘critical fat cell hypothesis’ that the achievement of a specific fat cell size triggers a subsequent increase in adipocyte number [22], [23]. Obese adipose tissues are formed in concert by both hypertrophy and hyperplasia of adipocytes. Several key molecules for adipose hyperplasia, such as serine-threonine kinase cyclin-dependent kinase 4 (Cdk4) [24], Cdk inhibitors p21 and p27 [25], and Skp2 [26], have been shown by using genetically-engineered animal models. These molecules also serve as a key regulator of cell cycle, e.g. Cdk inhibitors suppress cell proliferation while Skp2 accelerates cell cycle. Increases of fat weight and small number of adipocytes were observed in mice lacking p21 and p27 [25]. Skp2-deficient mice showed the decreases of fat weight and number of adipocytes, but the cell size of fat cells was not changed in Skp2-deficient mice [26]. Present study suggested that the increased Oip5 in obese adipose tissues might promote the proliferation of preadipocytes and accelerate the development of obesity. Insulin and TNF-α, those are augmented in obesity, increased Oip5 mRNA level, suggesting that such obesity-related signals increase the number of preadipocytes partly through Oip5 and provide new adipocytes for fat storage to maintain energy balance in a whole body.

Present study also demonstrated the proliferation of 3T3-L1 adipocytes by using EdU-staining and time-lapse imaging, which is consistent with a previous report that differentiated adipocytes were multiplied in *in vitro* [27]. Interestingly, Oip5 enhanced proliferation of not only preadipocytes but also the differentiated adipocytes, although adenovirus-mediated overexpression level of Oip5 protein was not determined. The useful antibody against murine Oip5 is not available at present and thus it will be needed in future to evaluate the protein expression level of Oip5 in vitro and in vivo. As shown in Figure 5, the *in vivo* adenovirus-mediated Oip5 overexpression resulted in the increase of cell number and fat weight. This result indicates that Oip5 may be one of key molecules for development of obesity, although it should be elucidated in future whether preadipocytes or adipocytes proliferated in the current *in vivo* adenovirus administration study. Multilocular adipocytes were partly observed in Ad-Oip5-injected subcutaneous fat tissues (Figure S6) and these fat cells were histologically similar to brown adipocytes. Adipose mRNA levels of UCP1, Cox7a1, and Cidea, which are markers for brown-like or beige adipocytes, were increased in Ad-Oip5-administered fat pads on Day 11 after adenovirus injection, but these increases were not statistically significant (data not shown). Several stimuli such as cold exposure, β3-adrenergic agonists, and PPARγ agonists, induce the brown-like adipocytes in white adipose tissues, so called ‘browning’ of WAT [28]–[33]. It would be clarified in future whether brown-like or beige adipocytes were induced in WAT by the acceleration of cell division via Oip5.

As demonstrated in Figure 6, adipose Oip5 was regulated by several factors. Oip5 mRNA level was gradually decreased during 3T3-L1 adipocytes differentiation (Day 2 to 9) while its level was elevated at adipocyte hypertrophic phase (Day 21). The latter may reflect the increase of Oip5 mRNA level in obese WAT as shown in Figure 1. However, it remains uncertain whether the increase of Oip5 mRNA level in Day 21 might be originated from the non-differentiated cells or the differentiated adipocytes. Interestingly, Oip5 mRNA level was significantly elevated by PPARγ agonists. PPARγ agonist was demonstrated to increase the number of small adipocytes and induce UCP1 in WAT [34], [35]. PPARγ agonist-induced increase of adipocytes may result in the amelioration of insulin resistance partly through augmentation of fat storage in adipocytes. As shown in Figure 5A, Ad-Oip5-injected subcutaneous fat tissues were histologically similar to PPARγ agonist-administered adipose tissues. There is a possibility that the effect of PPARγ activation on the adipocytes proliferation and the enlargement of fat cell size may be accounted for partly by the PPARγ-induced increase of Oip5. The *in vivo* physiological role of adipose Oip5 has not been clarified in present study because there were no significant metabolic changes in the DIO mice treated with Ad-Oip5. There is a possibility that adenovirus-mediated overexpression of Oip5 was transient and limited locally in fat tissues. Adipose-specific Oip5-transgenic and/or knockout animals will provide the significant role of adipose Oip5 in future.

In conclusion, Oip5 promotes proliferation of pre- and mature-adipocytes and contributes to adipose hyperplasia. Increase of Oip5 may accelerate development of obesity. Oip5 is expected as a new therapeutic target of obesity and type 2 diabetes.

## Supporting Information

Figure S1 **Protocol of knockdown for Oip5 study in 3T3-L1 preadipocytes.** 3T3-L1 preadipocytes were transfected with siRNA for Oip5-siRNA or Cont-siRNA. The transfected cells were incubated for 24 hrs and then reseeded to match the number of 3T3-L1 preadipocytes between Oip5-siRNA and Cont-siRNA groups. Bromodeoxyuridine (BrdU) was added at 22 hrs after reseeding and cell number was counted at 24, 48 or 72 hrs after reseeding.
(PDF)

Figure S2 **Protocol of Oip5 overexpression study in CAR-3T3-L1 preadipocytes.** 3T3-L1 cells stably expressing Coxsackie-Adenovirus Receptor (CAR-3T3-L1) were used in the adenoviral study. CAR-3T3-L1 preadipocytes were infected with adenovirus expressing Oip5 (Ad-Oip5) or adenovirus expressing β-galactosidase (Ad-βgal) at 5.0 multiplicity of infection (MOI). The medium was changed to remove the uninfected adenovirus at 24 hrs from adenovirus infection, and cells were reseeded to match the number of cells between Ad-Oip5 and Ad-βgal groups. Bromodeoxyuridine (BrdU) was added at 22 hrs after reseeding and cell number of CAR-3T3-L1 preadipocytes was measured at 24, 48 or 72 hrs after reseeding.
(PDF)

Figure S3 **Protocol of Oip5 overexpression study in CAR-3T3-L1 adipocytes.** CAR-3T3-L1 adipocytes were infected with Ad-Oip5 or Ad-βgal at 5.0 MOI on day 5 after differentiation into adipocytes. On 24 hrs after adenovirus infection, medium was changed to remove the uninfected adenovirus and CAR-3T3-L1 adipocytes were reseeded to match the number of adipocytes between Ad-Oip5 and Ad-βgal groups. CAR-3T3-L1 adipocytes were stained with Oil red O or subjected to cell counting at 24 or 72 hrs after reseeding. For the analysis of 5-ethynyl-2′-deoxyuridine (EdU) uptake, EdU was added to medium at 22 hrs after reseeding and CAR-3T3-L1 adipocytes were subjected to immunostaining at 24 hrs after reseeding.
(PDF)

Figure S4 **Correlation of OIP5 mRNA levels in peripheral blood cells and visceral fat area.** The study protocols and populations were previously described (Yamaoka M, Maeda N, Nakamura S, Kashine S, Nakagawa Y, et al. (2012) A pilot investigation of visceral fat adiposity and gene expression profile in peripheral blood cells. PLoS One 7:e47377.). The estimated visceral fat area (eVFA) was measured by abdominal bioelectrical impedance analysis (BIA), as reported previously (Ryo M, Maeda K, Onda T, Katashima M, Okumiya A, et al. (2005) A new simple method for the measurement of visceral fat accumulation by bioelectrical impedance. Diabetes Care 28∶451–453). Briefly, blood total RNA samples were obtained from 28 subjects (BMI 31.9±6.0 kg/m^2^, eVFA 199.4±89.4 cm^2^) and were subjected to Agilent whole human genome 4×44 K oligo-DNA microarray (Agilent Technologies, Santa Clara, CA). The raw microarray data are deposited in the National Center for Biotechnology Information Gene Expression Omnibus (GEO Series GSE28038). The correlation between peripheral blood OIP5 mRNA level and Log-eVFA levels was examined by Pearson’s correlation under the R environment (R^2^ = 0.2559, *P* = 0.0060).
(PDF)

Figure S5 **Tissue distribution of Oip5 mRNA level in mice.** C57BL/6N mice were analyzed under 12 hrs-fasting state at 12 weeks of age. BAT, brown adipose tissues; Sub WAT, subcutaneous white adipose tissues; Epi WAT, epididymal white adipose tissues.
(PDF)

Figure S6 **Representative higher magnification of hematoxylin and eosin (H&E)-staining section of fat tissues on day 11 after adenovirus injection.** Eight-week-old male C57BL/6N mice were made incisions at approximately 1 cm in bilateral groins and the 25 µL (2.5×10^9^ pfu/mL) of Ad-βgal and Ad-Oip5 were injected in each side of the subcutaneous fat at the groin, respectively. Shown are representative hematoxylin and eosin (H&E) stainings on day 11 after adenovirus injection. Oip5, Opa-interacting protein 5; βgal, β-galactosidase.
(PDF)

Movie S1 **Time-lapse imaging for living adipocytes.** CAR-3T3-L1 adipocytes were reseeded on 2 chamber dishes at 24 hrs after adenovirus infection and on day 7 after differentiation adipocytes were subjected to time-lapse imaging from 24 to 44 hrs after reseeding. A sealed dish of CAR-3T3-L1 adipocytes was placed in an acrylic resin box in which temperature was maintained at 37°C on the sample stage, and time-lapse images were captured every 6 min. Arrors indicate the division of adipocytes. Scale bar = 30 µm.
(MOV)

Movie S2 **Time-lapse imaging for living adipocytes.** CAR-3T3-L1 adipocytes were reseeded on 2 chamber dishes at 24 hrs after adenovirus infection and on day 7 after differentiation adipocytes were subjected to time-lapse imaging from 24 to 44 hrs after reseeding. A sealed dish of CAR-3T3-L1 adipocytes was placed in an acrylic resin box in which temperature was maintained at 37°C on the sample stage, and time-lapse images were captured every 6 min. Arrors indicate the division of adipocytes. Scale bar = 30 µm.
(MOV)
