# Supplementary figures and images for: Possible Involvement of Opa-Interacting Protein 5 in Adipose Proliferation and Obesity
Source: PLoS One. 2014 Feb 6;9(2):e87661. doi: 10.1371/journal.pone.0087661 (PMC3916335; doi:10.1371/journal.pone.0087661)

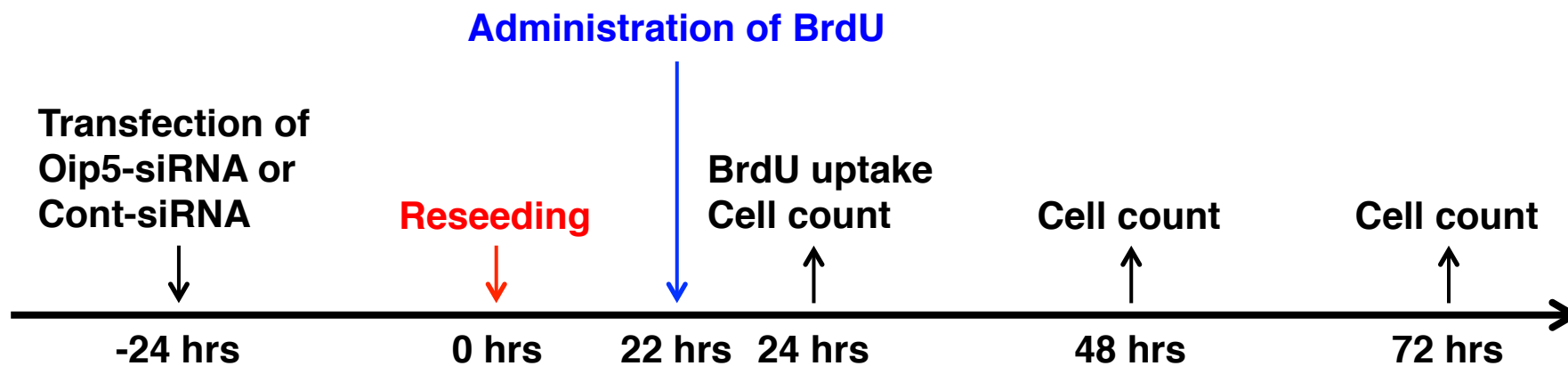

**3T3-L1 preadipocytes**

**Figure S1**

Supplement: Figure S1 — Protocol of knockdown for Oip5 study in 3T3-L1 preadipocytes. 3T3-L1 preadipocytes were transfected with siRNA for Oip5-siRNA or Cont-siRNA. The transfected cells were incubated for 24 hrs and then reseeded to match the number of 3T3-L1 preadipocytes between Oip5-siRNA and Cont-siRNA groups. Bromodeoxyuridine (BrdU) was added at 22 hrs after reseeding and cell number was counted at 24, 48 or 72 hrs after reseeding. (PDF) [file pone.0087661.s001.pdf]

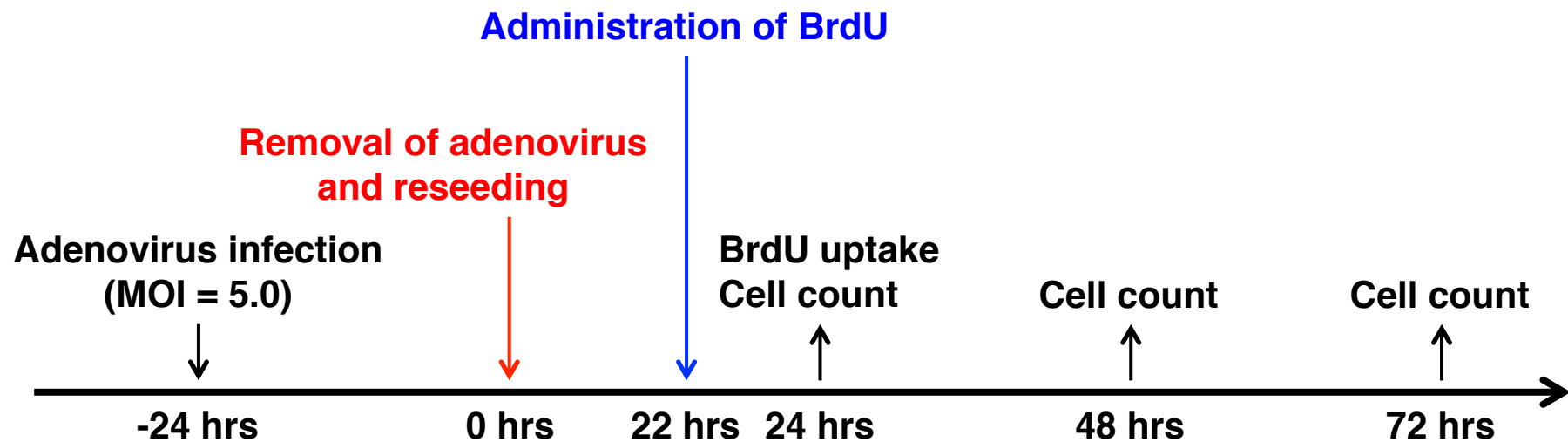

**CAR-3T3-L1 preadipocytes**

**Figure S2**

Supplement: Figure S2 — Protocol of Oip5 overexpression study in CAR-3T3-L1 preadipocytes. 3T3-L1 cells stably expressing Coxsackie-Adenovirus Receptor (CAR-3T3-L1) were used in the adenoviral study. CAR-3T3-L1 preadipocytes were infected with adenovirus expressing Oip5 (Ad-Oip5) or adenovirus expressing β-galactosidase (Ad-βgal) at 5.0 multiplicity of infection (MOI). The medium was changed to remove the uninfected adenovirus at 24 hrs from adenovirus infection, and cells were reseeded to match the number of cells between Ad-Oip5 and Ad-βgal groups. Bromodeoxyuridine (BrdU) was added at 22 hrs after reseeding and cell number of CAR-3T3-L1 preadipocytes was measured at 24, 48 or 72 hrs after reseeding. (PDF) [file pone.0087661.s002.pdf]

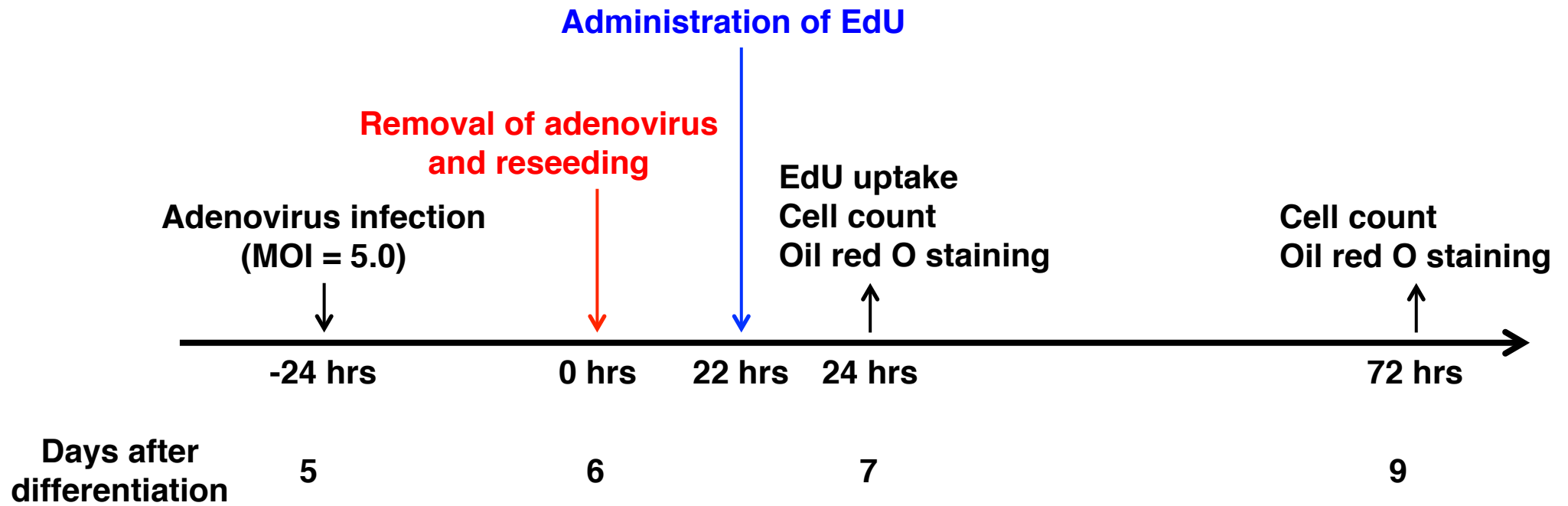

**CAR-3T3-L1 adipocytes**

**Figure S3**

Supplement: Figure S3 — Protocol of Oip5 overexpression study in CAR-3T3-L1 adipocytes. CAR-3T3-L1 adipocytes were infected with Ad-Oip5 or Ad-βgal at 5.0 MOI on day 5 after differentiation into adipocytes. On 24 hrs after adenovirus infection, medium was changed to remove the uninfected adenovirus and CAR-3T3-L1 adipocytes were reseeded to match the number of adipocytes between Ad-Oip5 and Ad-βgal groups. CAR-3T3-L1 adipocytes were stained with Oil red O or subjected to cell counting at 24 or 72 hrs after reseeding. For the analysis of 5-ethynyl-2′-deoxyuridine (EdU) uptake, EdU was added to medium at 22 hrs after reseeding and CAR-3T3-L1 adipocytes were subjected to immunostaining at 24 hrs after reseeding. (PDF) [file pone.0087661.s003.pdf]

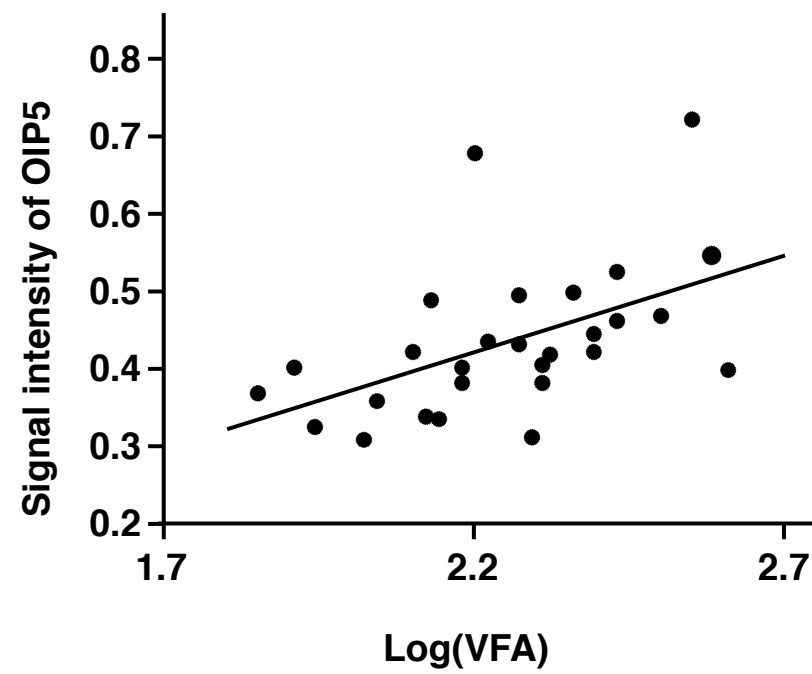

**Figure S4**

Supplement: Figure S4 — Correlation of OIP5 mRNA levels in peripheral blood cells and visceral fat area. The study protocols and populations were previously described (Yamaoka M, Maeda N, Nakamura S, Kashine S, Nakagawa Y, et al. (2012) A pilot investigation of visceral fat adiposity and gene expression profile in peripheral blood cells. PLoS One 7:e47377.). The estimated visceral fat area (eVFA) was measured by abdominal bioelectrical impedance analysis (BIA), as reported previously (Ryo M, Maeda K, Onda T, Katashima M, Okumiya A, et al. (2005) A new simple method for the measurement of visceral fat accumulation by bioelectrical impedance. Diabetes Care 28∶451–453). Briefly, blood total RNA samples were obtained from 28 subjects (BMI 31.9±6.0 kg/m2, eVFA 199.4±89.4 cm2) and were subjected to Agilent whole human genome 4×44 K oligo-DNA microarray (Agilent Technologies, Santa Clara, CA). The raw microarray data are deposited in the National Center for Biotechnology Information Gene Expression Omnibus (GEO Series GSE28038). The correlation between peripheral blood OIP5 mRNA level and Log-eVFA levels was examined by Pearson’s correlation under the R environment (R2 = 0.2559, P = 0.0060). (PDF) [file pone.0087661.s004.pdf]

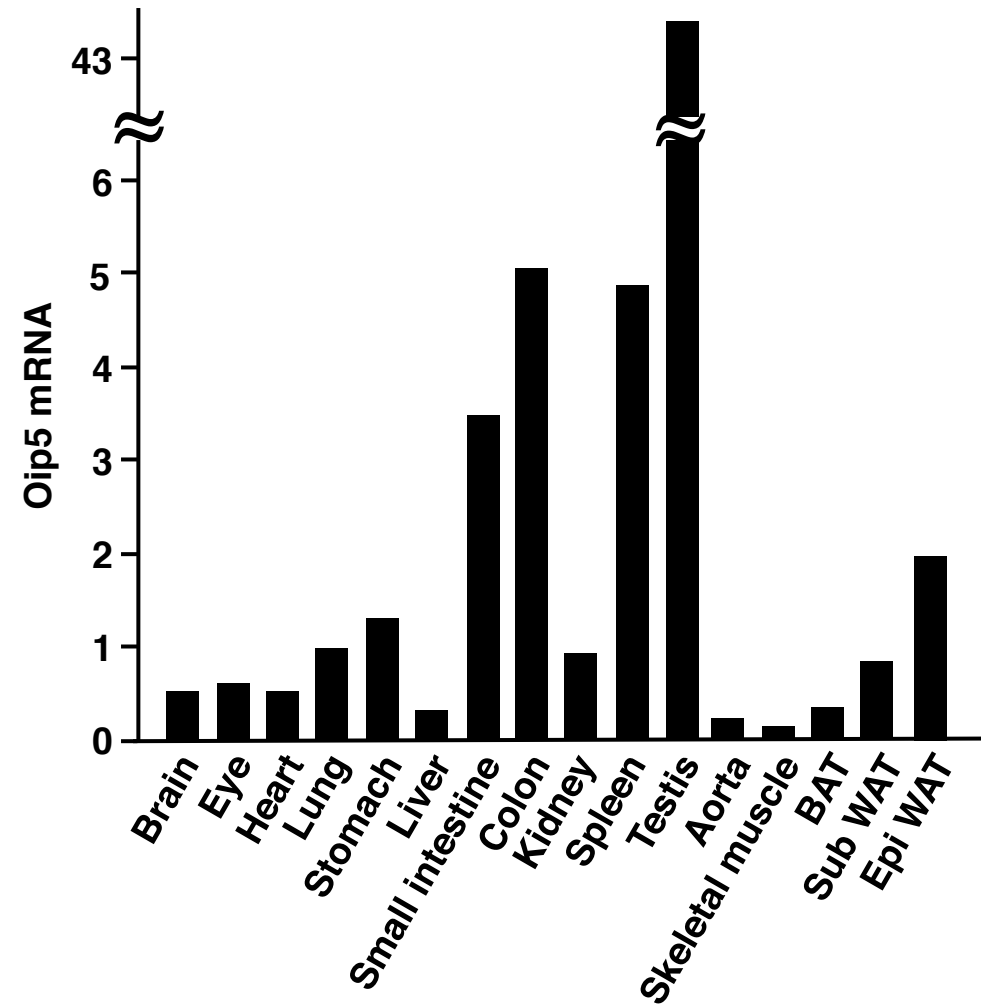

Figure S5

Supplement: Figure S5 — Tissue distribution of Oip5 mRNA level in mice. C57BL/6N mice were analyzed under 12 hrs-fasting state at 12 weeks of age. BAT, brown adipose tissues; Sub WAT, subcutaneous white adipose tissues; Epi WAT, epididymal white adipose tissues. (PDF) [file pone.0087661.s005.pdf]

**$\beta$ gal**

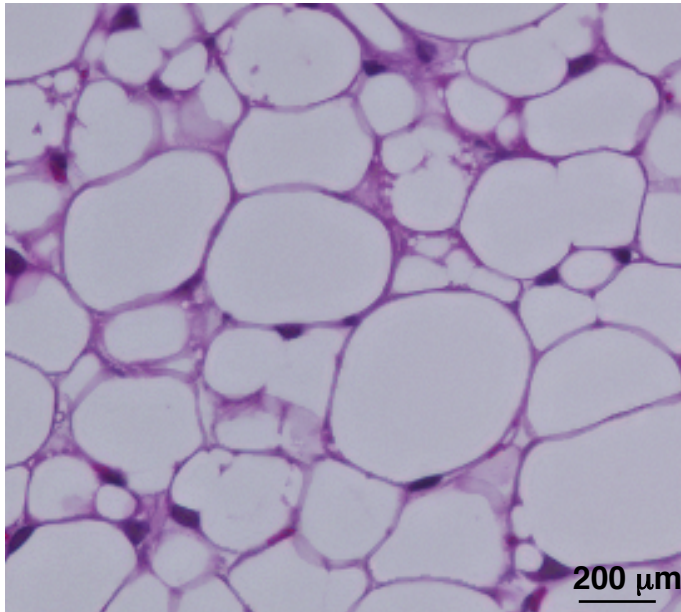

**Oip5**

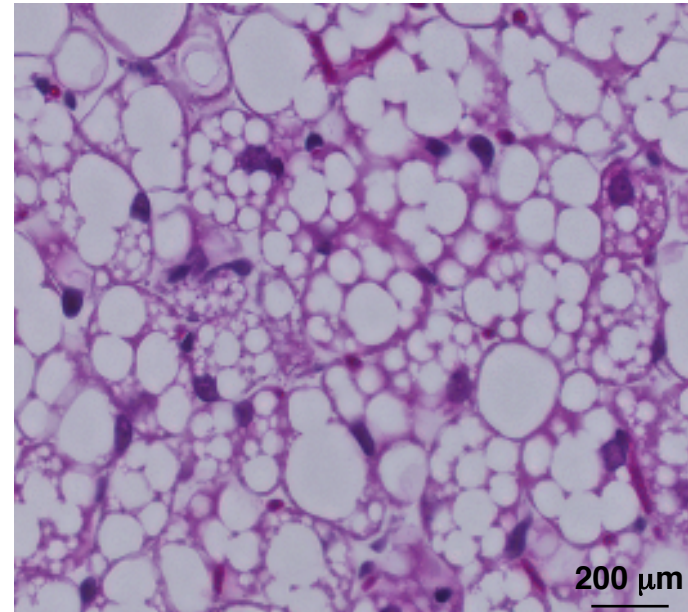

**Figure S6**

Supplement: Figure S6 — Representative higher magnification of hematoxylin and eosin (H&E)-staining section of fat tissues on day 11 after adenovirus injection. Eight-week-old male C57BL/6N mice were made incisions at approximately 1 cm in bilateral groins and the 25 µL (2.5×10^9^ pfu/mL) of Ad-βgal and Ad-Oip5 were injected in each side of the subcutaneous fat at the groin, respectively. Shown are representative hematoxylin and eosin (H&E) stainings on day 11 after adenovirus injection. Oip5, Opa-interacting protein 5; βgal, β-galactosidase. (PDF) [file pone.0087661.s006.pdf]
